# Veterinary Medical Society of the University of Pennsylvania

**Published:** 1898-01

**Authors:** M. Jacob

**Affiliations:** Secretary


### VETERINARY MEDICAL SOCIETY OF THE UNIVERSITY OF PENNSYLVANIA.

Meeting called to order at 8 P.M., December 17, 1897, in the College amphitheatre. Mr. Spaeth was appointed critic, and Messrs. Johnson, Miller, and Nolan were appointed judges of the debate. Mr. Horner, '98, read a very interesting paper on "The Care of Milk on the Farm." Mr. Cheney, '99, gave an excellent talk on "What Horses are Considered the Origin of Trotting-blood in this Country."

Next in order was the debate: " Resolved, That Philadelphia is in need of a better system of meat-inspection." Affirmative: Mr. Kirby, '98; Mr. Keiter, '99; Mr. Phillips, 1900. Negative: Mr. McClure, '98; Mr. Hoopes, '99; Mr. Reardon, 1900. The judges decided in favor of the negative; nevertheless some good arguments were presented on both sides, and special mention must be made of the display of oratory presented by Mr. McClure, as his powers as a debater and orator are surpassed by few.

After the debate the Society adjourned and prepared for the banquet, which was held at the University Restaurant by the Veterinary Medical Society. The members of the Faculty present were Professors Pearson, Adams, Harger, and Hoskins, also the Resident Surgeon, Dr. Shaw. The attendance of the students was large, and the magnificent supper seemed to be enjoyed by all present. After supper several addresses were made by the professors and other members present, which made the occasion a very interesting one, and when the hour of departure came every one seemed well pleased with the proceedings of the evening, and without doubt a banquet will henceforth be an annual affair of this Society.

The last two or three meetings of the Society have been of special interest, for two reasons: first, by the zeal which the members show in their parts, and secondly, by the nature of the papers read and by the questions debated. Some of the papers read were as follows: " The Ox-bot, and How it Affects Cattle in the West and Southwest," by Mr. Blount, class '98. " Why Enter the Veterinary Profession," Mr. Repp, '98. Jersey Cattle as a Dairy Breed," Mr. Hoopes, '99. " Consumption in Cattle Conveyable to Man," by Mr. Spindler," '98. " How a Lebanon Veterinarian Reduced a Hernia on the Abdomen of a Horse," Mr. Miller, '99.

Some of the questions debated were: *Resolved*, " That the requirements for entrance into the veterinary college should be raised." *Resolved*, " That the preservation of public health depends more upon the veterinarian than upon the practitioner of human medicine." *Resolved*, " That the State should recompense the owners of glanderous horses to 50 per cent. of their healthy value."

Beside the foregoing there were also several very good addresses made by members of the Faculty. At one of the recent meetings the Librarian informed the Society that Prof. Adams had presented the Society with the *Veterinary Magazine* for three years.

A very good measure brought about by the Society was the prohibiting of strangers in the operating-room during operations.

At the next election of officers one of the most important changes will be the election a new Honorary President in the place of Prof. R. S. Huidekoper, and the new officer will probably be one of the members of the Faculty. At a recent meeting the name of Dr. Megary was proposed, and he has duly elected as honorary member of the Society.

The first meeting after the holiday vacation will take place in the lower lecture-room, Friday evening, January 7, 1898, at 7.30 o'clock. The programme promises to be an interesting one, and all are cordially invited to be present.

M. JACOB,
Secretary.

